# GP participation in increasing uptake in a national bowel cancer screening programme: the PEARL project

**DOI:** 10.1038/bjc.2017.129

**Published:** 2017-05-18

**Authors:** Sally C Benton, Piers Butler, Katy Allen, Michelle Chesters, Sally Rickard, Sally Stanley, Richard Roope, Daniel Vulkan, Stephen W Duffy

**Affiliations:** 1 NHS Bowel Cancer Screening Programme Southern Hub, Surrey Pathology Services, 20 Priestley Road, Surrey Research Park, Guildford, Surrey, GU2 7YS, UK; 2 Department of Biochemistry and Physiology, University of Surrey, Guildford, Surrey GU2 7XH, UK; 3 Wessex Cancer Clinical Network, Oakley Road, Southampton, Hampshire SO16 4GX, UK; 4 The Whiteley Surgery, Yew Tree Drive, Whiteley, Fareham PO15 7LB, UK; 5 Centre for Cancer Prevention, Wolfson Institute of Preventive Medicine, Queen Mary University of London, London EC1M 6BQ, UK

**Keywords:** colorectal neoplasia, screening, uptake, faecal occult blood test, compliance, general practice, endorsement

## Abstract

**Background:**

The NHS Bowel Cancer Screening Programme (BCSP) in England does not involve general practitioners (GPs). Uptake is ∼58%. The Practice Endorsed Additional Reminder Letter (PEARL) study piloted a GP-endorsed reminder letter.

**Methods:**

General practices in Wessex with uptake <55% (prevalent invitations) were invited to participate. Subjects who had been invited for screening, sent a standard 28-day BCSP reminder letter but had not returned a test kit within 30 days of the standard reminder were sent a second reminder letter bearing the GP’s letterhead and signature. Uptake was compared between PEARL and non-PEARL practices by standardised uptake ratio (standardised for prior prevalent uptake and other confounders). In addition, 25 non-PEARL practices were matched with PEARL practices for prior prevalent uptake and number of invitees.

**Results:**

Twenty-five practices agreed to participate. A total of 3149 GP-endorsed reminders were sent. Uptake in the PEARL practices was 54% compared with 51% in the matched-control practices. The adjusted RR for uptake was 1.08 (95% CI: 1.05, 1.11, *P*<0.001) for all invitees and 2.18 (1.79, 2.66, *P*<0.001) for invitees who had not returned a kit following the standard reminder.

**Conclusions:**

The GP-endorsed reminder was associated with significantly increased uptake among subjects not responding to the standard reminder letter.

Worldwide, colorectal cancer (CRC) is a major health burden; in 2012, nearly 1.4 million cases were diagnosed and there were 694 000 deaths (Ferlay *et al*, 2013). Screening using guaiac-based faecal occult blood testing (gFOBt) reduces mortality from CRC, as demonstrated by several population-based randomised controlled trials (Mandel *et al*, 1993; Kewenter *et al*, 1994; Hardcastle *et al*, 1996; Kronborg *et al*, 1996). The Bowel Cancer Screening Programme (BCSP) was implemented by the National Health Service (NHS) in England in 2006 and is delivered by five regional Hubs. Men and women aged 60–74 years (inclusive) receive an invitation to participate in screening using gFOBt every 2 years. The initial stage of the process is a pre-invitation letter that explains the disease and how the screening programme works. A week later, a gFOBt kit is sent out with another invitation letter. All correspondence from the Hub is signed by the BCSP Hub Director, who is most likely unknown to the subject receiving the letter due to the large geographical regions covered by each Hub.

The gFOBt requires participants to smear two small faecal samples from each of three separate stools onto a card that, when complete, is returned in the post to the BCSP Hub for analysis. Participants that receive a positive (abnormal) test result are referred for a follow-up investigation; if the test is negative (normal), participants will be invited to be screened again in 2 years’ time until they reach the age of 75 (after which time individuals may self-refer). A reminder letter is sent to subjects where no action has occurred on the Bowel Cancer Screening System within 28 days of being sent a test kit. Screening episodes are closed if there is no response 17 weeks after the kit was sent.

In England, ‘uptake’ of screening is defined as the proportion of invited subjects that is adequately screened (definitive positive or definitive negative test result) within 26 weeks of invitation. Overall uptake in England was 58.2% in 2014/2015 (personal communication, National Office), considerably lower than in the breast and cervical screening programmes (Douglas *et al*, 2016. There is wide variation in uptake across the country. BCSP data have demonstrated uptake to be as low as 35% in the most deprived areas and as high as 63% in least deprived areas (von Wagner *et al*, 2011).

There is evidence to suggest that people are more likely to respond to an invitation to screening if they are encouraged to do so by their GP or another health professional (Cole *et al*, 2002; Senore *et al*, 2010; Zajac *et al*, 2010; Hewitson *et al*, 2011; Camilloni *et al*, 2013; Shankleman *et al*, 2014; Massat *et al*, 2014; Hall *et al*, 2015; Wardle *et al*, 2015).

The aim of the Practice Endorsed Additional Reminder Letter (PEARL) project was to develop and pilot a sustainable intervention to engage subjects in bowel cancer screening. The project was designed to test whether an additional reminder letter on GP-headed paper and with the signature of the subject’s GP could encourage uptake. It is the result of collaboration between the BCSP Southern Hub, a group of GPs in Wessex (Hampshire, Dorset and the Isle of Wight) working for Macmillan Cancer Support and Cancer Research UK, in partnership with the Wessex Strategic Clinical Network.

## Materials and methods

### Target population

The BCSP Southern Hub serves the south of England (excluding London), sending 20 000 invitations for screening every week. The PEARL project tested the intervention in the target area of Wessex. All general practices in Wessex with an uptake of less than 55% (‘prevalent’ episodes only and calculated for all screening data up to 10 September 2013) were invited to participate. These practices were typically in areas of socioeconomic deprivation. The PEARL intervention targeted all subjects aged 60–74 years (inclusive), registered with a participating practice who were invited to take part in screening and had not responded to the standard 28-day BCSP reminder letter.

### Recruitment and participants

Eligible practices were contacted by e-mail and regular mail to invite them to participate. If no reply was received, they were contacted by telephone. The deadline for agreement to participate was 31 May 2014. Participating practices agreed to receive an educational 1-h visit to learn about the BCSP, the PEARL project and to discuss the practice-specific statistical cancer profile—the National Cancer Intelligence Network general practice profile. Practices were offered a financial incentive (£250) for participating in the project.

Each practice signed a consent form and a confidentiality agreement and data exchange form, which set out the responsibilities of both the Hub and the GP practice (Supplementary web-Appendix A).

The text of a standard second reminder letter (Supplementary web-Appendix B) was approved by the BCSP National Office.

Each participating practice sent the Hub a paper or scanned electronic copy of the practice letterhead and a digital GP signature. One GP signed the letter for all patients from the practice. An electronic letter template was constructed for each practice.

### Method

**Figure 1:**
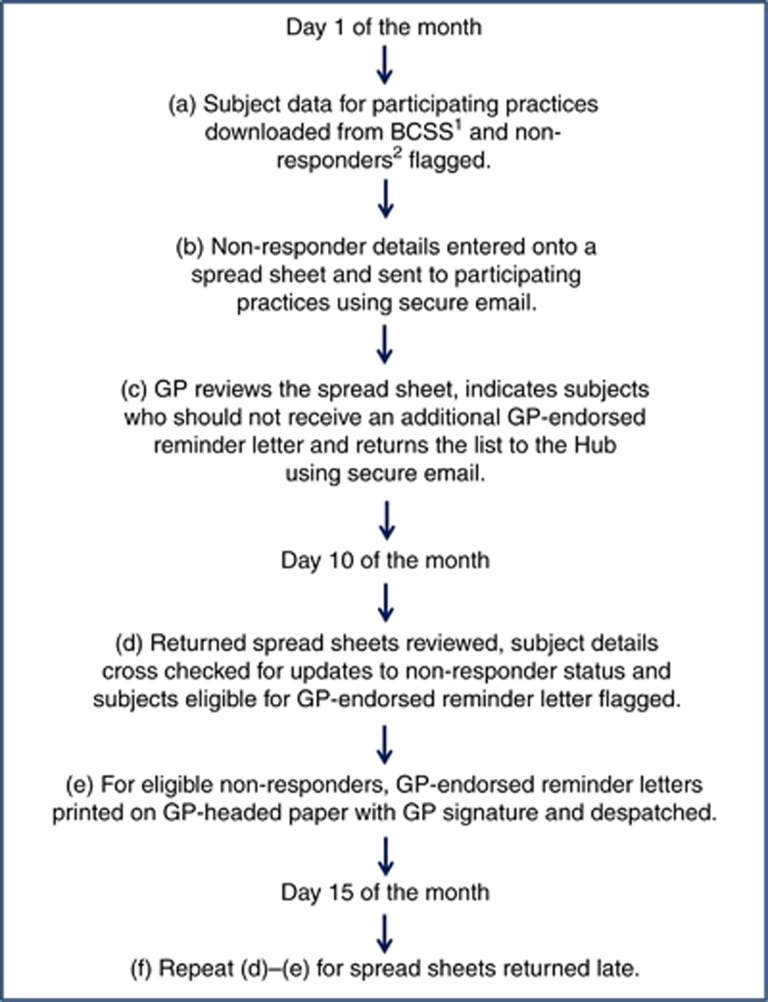
**Schematic representation of PEARL process.** ^1^BCSS: Bowel Cancer Screening System; ^2^Non-responders defined as subjects who had not returned a test kit within 30–60 days of a standard reminder letter.

The PEARL intervention took place in Wessex between September 2014 and October 2015 and was designed to run for a fixed period of time. The flowchart in Figure 1 summarises the PEARL process. On the first Wednesday of each month, BCSP subject-level data (subject name, address, number of test kits sent, number of test kits returned, date of initial invitation, date standard 28-day reminder letter sent) for each participating practice were downloaded from the ORACLE-based BCSP database (Bowel Cancer Screening System). The full name, NHS number, and address of all non-responders whose kit had been sent out 60–90 days prior to the extract date were entered onto a spreadsheet for each practice and sent using secure mail. The broad 60–90-day range is due to the programme download being run on a single day each month, and using a 30-day moving window to ensure no subjects were missed. The practice reviewed each subject’s history and indicated on the spreadsheet using a drop-down menu whether the subject should be sent an additional reminder.

GPs returned completed spreadsheets to the Hub by secure e-mail.

Ten working days after the initial list was sent, the list of subjects on returned GP spreadsheets was checked against a new download of subject-level data from Bowel Cancer Screening System to ensure that subjects remained non-respondent, were still registered with the same GP, and that there was no record of death. GP-endorsed reminder letters were printed for all eligible subjects with the appropriate GP letterhead and signature. This process was repeated 15 working days after the initial list for any GP spreadsheets returned late.

In any 1 month, if no non-responders were identified for a participating practice, the practice was notified by e-mail that they would not receive a list that month. Practices that did not return a completed spreadsheet were sent a reminder e-mail 5 working days after the spreadsheet was sent.

The average date of sending a GP-endorsed reminder was 99 days after initial invitation. Subjects in PEARL-registered practices not sent a reminder and in the non-PEARL practices were assigned an index date, the date when a PEARL reminder would have been sent if the practices were participating and the subject had not yet returned an adequate kit. Uptake, the primary outcome measure, was defined as the proportion of subjects (of the overall invited population and of those not adequately screened before the index date) that was adequately screened. Uptake was compared between PEARL practices and all other practices in the Southern Hub region with a prevalent uptake of less than 55%. The comparison group included all routine screened Southern Hub subjects with an invitation on days that PEARL subjects were invited.

Subject variables were sex, age-group, and screening history (categorised as ‘prevalent’ first-time invitee or previous non-responder or ‘incident’ previous responder, plus sequence of episode—first, second, third, etc). In addition, a measure of social deprivation (index of multiple deprivation (IMD)) was assigned for each subject with reference to the English indices of deprivation. The IMD scores were grouped into quintiles based on national distributions with the use of predefined national cutoffs.

### Statistical methods

Both the 25 PEARL-registered practices and the 1575 comparison practices were specified to have prevalent screen participation rates of less than 55% prior to the intervention. However, even within this range, the comparison practices had substantially higher prior participation rates than the PEARL-registered practices (see Results section). We therefore analysed the data analogously to standardised incidence or mortality ratios. Using logistic regression, in subjects within the comparison practices only, we estimated the effects of age, sex, prior prevalent participation, IMD quintile, episode type (prevalent or incident), and episode sequence on the probability of participation. These were applied to the subjects in the PEARL-registered practices to calculate the expected proportion of participants in these practices (see Supplementary web-Appendix C for mathematical details). The effect of the intervention was estimated as the observed number of participants in the PEARL-registered practices divided by the expected number. We calculated confidence intervals on the ratio assuming a binomial distribution of numbers participating, taking into account the uncertainty in the expected numbers.

For subjects in the non-PEARL-registered practices, the index date was defined as the date on which a PEARL reminder would have been due if these had been PEARL subjects. Our primary analysis was to compare the participation rate of subjects in the PEARL-registered practices with that expected from the comparison practices, for overall participation and for participation in those not returning an adequate kit by the index date, unstratified by demographic or screening data. Secondary analysis consisted of the same comparisons in subgroups of age, sex, screening episode type (prevalent or incident), and IMD quintile.

As a check on our method, we also selected 25 non-PEARL practices, matched to the PEARL-registered practices by previous prevalent participation rates and number of invitees. We repeated our primary analysis, directly comparing participation rates in PEARL-registered practices with those in the matched non-PEARL practices. Analysis for this comparison was performed using inverse variance weighted average estimation conditioning on matched set (Cummings, 2009). All analyses were performed in Stata version 13.1 (StataCorp, 2013).

### Funding and ethics

The PEARL project protocol was approved by the NHS BCSP Research Committee in March 2015 (reference ID 148). The Office for Data Release granted permission for sharing BCSP data with Professor Stephen Duffy (statistician) in November 2015 (reference ODR1516_154). Patient consent was not required under Section 251 of the 2006 NHS Act. The PEARL project is part of the ACE (Accelerate, Coordinate, Evaluate) programme, NHS England’s initiative supported by Cancer Research UK and Macmillan Cancer Support to diagnose cancer early and improve outcomes. Funding was received from the National Cancer Intelligence Network.

## Results

Of the 43 eligible practices approached, 25 agreed to participate in the PEARL study. Over the 14-month study period, 324 non-participating subject lists were sent to GP practices. Seventeen practices had lists to review every month. For 11 of the study months, there were practices (*n*=1–7) that received no list. The mean number of subjects on each list was 16.02 (range 1–103).One hundred twenty-four (38.3%) lists were not returned. Two of the practices that signed up to the PEARL intervention did not return any of the lists.

One hundred sixty-one (5.11%) subjects were deemed unsuitable by the GP to receive a reminder letter for the following reason:

- Patients receiving end-of-life care (*n*=10)
- Already having regular colonoscopy surveillance (*n*=20)
- Recent (<12 months) diagnosis of bowel cancer (*n*=4)
- Unable to have colonoscopy (*n*=5)
- Other (*n*=122).

There were 12 878 invitees in the PEARL-registered practices and 1 248 689 in the non-PEARL-registered practices. A total of 3149 GP-endorsed reminder letters were sent.

Table 1 shows the characteristics of subjects in the PEARL-registered practices, in those practices matched to the PEARL practices, and in all the comparison practices. There were substantial differences between the invitees in the PEARL-registered practices and those in the non-PEARL-registered practices. In particular, the former were much more likely to be in deprived IMD categories. This is reflected in the fact that average prior prevalent participation in the PEARL-registered practices was 36%, substantially lower than that observed in the non-PEARL practices, 47%. This necessitated the approach described above, deriving results standardised for age, sex, IMD quintile, episode type, episode sequence, and prior prevalent participation. The differences between the PEARL practices and the matched practices are much attenuated, and the average prior prevalent participation in the matched practices was 36%, as in the PEARL-registered practices. Table 2 shows the numbers returning a completed kit by the index date in the same groups.

Table 3 shows the basic results with respect to participation. There was a highly significant (*P*<0.001) difference between the participation rates in the PEARL practices and that expected from the non-PEARL, with 6914 (54%) participation *vs* 6543 (51%) expected. In those who qualified for a reminder, that is, those who had not returned a completed kit by the index date, there was also a highly significant difference (*P*<0.001) with 362 (7%) participation in the PEARL practices compared with 167 (3%) expected. The direct comparison of the 25 PEARL practices with the 25 matched practices showed slightly stronger but essentially similar results (Table 4).

Table 5 shows the subgroup results by age, sex, screen type (prevalent/incident), and socioeconomic status as measured by IMD quintile, for all invitees, and for those not returning a completed kit by the index date. In the upper half of the table, all invitees, it can be seen that significantly greater participation than expected was noted in the PEARL practices for all subgroups except for subjects aged 65 years or less, with absolute differences ranging from 2 to 5%. The difference in the effect of the PEARL intervention between age groups was statistically significant (*P*=0.001). No other significant heterogeneity of the PEARL intervention effect between subgroups was observed.

In the corresponding results in the lower half of the table, for those with no return of an adequate kit before the index date, all subgroups showed highly significant results, with an approximate doubling of the rate of participation, typically from 2–3 to 6–8%. Again, there was significant heterogeneity of the effect by age, with a stronger effect of the PEARL intervention in those older than 65 years. No other significant heterogeneity of the intervention effect was observed between subgroups.

Using the method and rates given by Raine *et al* (2016), we predict the effect of increased uptake of screening from PEARL on observed outcomes nationally. In the 2014/2015 fiscal year, 4 117 866 people were invited for screening by the BCSP in England. An increase of 3% suggests that if PEARL was implemented nationally then an extra 123 536 each year would be screened. In 2014/2015, the positivity rate among the screened population was 1.79%, and 87.39% of these attend a specialist screening practitioner clinic. In all, 8.09% will have a colorectal cancer and 23.43% will have medium- or high-risk polyps. Hence, if PEARL were implemented nationally, this could detect up to an additional 453 people (123536*0.0179*0.8739*0.2343) with high- or intermediate-risk polyps, and 156 people (123536*0.0179*0.8739*0.0809) with a colorectal cancer each year.

## Discussion

Our results indicate that the addition of a GP-endorsed reminder at 3 months significantly increased participation in the NHS BCSP, by 3% in absolute terms (54% *vs* 51%), in general practices with participation rates below the national average. There is already evidence that primary care endorsement improves participation in screening for a number of cancers (Duffy *et al*, 2016) and this study provides further evidence to support this.

Our aim was to develop a robust, feasible, and sustainable method to increase uptake through direct GP endorsement of the BCSP in England. Although previous studies have demonstrated that GP endorsement is successful in increasing uptake in the programme (Cole *et al*, 2002; Senore *et al*, 2010; Zajac *et al*, 2010; Hewitson *et al*, 2011; Camilloni *et al*, 2013), these studies have not considered the long-term feasibility of uptake initiatives directly involving GP practices and, to our knowledge, none has targeted non-responders and asked GPs to assess the appropriateness of sending a subject a reminder letter.

Hall *et al* (2015) conducted a qualitative study using in-depth interviews with BCSP non-participants and found that non-participation in screening was not necessarily associated with negative attitudes towards screening or a decision not to return a kit. The authors concluded that some non-participants may have a degree of intention to take part in screening in the future and may be more responsive to interventions based on professional endorsement and reminders. The results from our study support this.

Another RCT was conducted in the south of England in 2009 among 20 general practices (1288 subjects invited for screening) (Hewitson *et al*, 2011). Subjects were randomised to either a GP-endorsed letter and/or an enhanced information leaflet with their gFOBT kit. The GP-endorsed letter and the enhanced procedural leaflet increased uptake by 5.8% and 6.0%, respectively, and had an additive effect (11.8%).

Although we have made the assumption that it is the GP endorsement that has led to our observed increase in uptake, due to the design of our study we cannot be certain whether an additional reminder letter from the Hub would in itself increase uptake in the programme. As summarised by Duffy *et al* (2016), reminders have been demonstrated to increase uptake in screening programmes. However, only two studies have reported the effect of postal reminders alone on participation, one of pre-appointment breast screening reminders (Allgood *et al*, 2016) and one of reminders following non-attendance in cervical screening (Eaker *et al*, 2004). Although both studies found an increase in participation with the reminders, it is not clear whether these results would generalise to the Bowel Cancer Screening Programme in the UK. To our knowledge, no studies assessing the impact of a second reminder letter have been published. In our primary standardised analysis, the effect of the intervention in terms of raw numbers was to increase participation by 371 (6914–6543) when all invitees are considered. Restricting analysis to those who had not returned an adequate kit by the index date (the target population of the intervention), the effect was an increase of 195 (362–167). In the matched analysis, the figures would be 512 (6914–6914/1.08) (Table 4) and 196 (362–362/2.18), respectively. One could therefore be confident that the effect of adopting these reminders as policy would lie within the range of these figures. In terms of the larger number of participants estimated to accrue when all invitees are considered, it is not out of the question that the intervention had some effect on participation among those not sent a PEARL reminder but living with a subject who did receive a reminder.

During the intervention period, 3149 GP-endorsed reminders were sent in the PEARL-registered practices. The resulting absolute increase in participation was of the order of 200 in the target population. This is consistent with the observation that of those adequately screened, but not before the index date, in the PEARL-registered practices, 250 actually received a reminder. For each extra participant, between 6 and 16 PEARL reminders had to be sent, depending on which estimate one considers. The PEARL-registered practices were characterised by low socioeconomic status (Table 1), therefore the intervention has potential to improve delivery to traditionally underserved populations.

A limitation of this study is that the intervention was not randomised. However, we did include in the PEARL practices those which registered whether or not they supplied lists, to approximate an intention to treat analysis. Also, the similar results by two separate approaches, taking account of differences between the PEARL practices and the comparison practices, give some confidence in the results.

Two practices that had agreed to take part in the PEARL project did not return any non-responder patient lists but the practices may still have used the information to target patients—it is not possible to quantify that effect. The exclusion of non-responders by GPs could not be replicated in the control population, but this limitation would be likely to diminish the observed effect of the intervention. The process is dependent on engagement and active participation of GP practices and our experience demonstrated that logistical factors such as use of a generic e-mail address, key staff members leaving can impact the success of such projects.

The Hub process to generate the GP-endorsed second reminder letter was robust and became incorporated into the routine workload of the Southern Hub. For the majority of subjects, the fact the letter was signed by the subject’s own GP practice and appeared on the GP’s letterhead (as opposed to a letter signed by an unknown Hub Director) is likely to have had a positive effect on the subject’s attitude. Other research has demonstrated that uptake of screening can be improved by direct contact in person with a GP or other health professional (Senore *et al*, 2010), especially in ethnically diverse populations (Shankleman *et al*, 2014; Massat *et al*, 2014), although such an approach has important resource implications in terms of cost and time and is probably unsustainable.

The results of this project require qualification, not least the labour-intensive method and high level of quality control required. Despite offering a financial incentive, in addition to the potential benefit to their patients, only 58% of invited GP practices agreed to participate. Anecdotal reasons given by practices for not participating in the PEARL project included the amount of time that would have to be invested, an unusually high turnover of practice managers, and organisational disruption caused by practice mergers during the study period.

The reasons given above for non-participation should be interpreted with caution, The practices invited to take part were those with the lowest uptake (<55% for prevalent invitations) and the link between deprivation and low uptake of bowel cancer screening has been firmly established. It may be that the observations should not be extrapolated to all GP surgeries on a national scale.

A key consideration for the study was the GP–patient relationship if a letter was coming on GP letter headed paper. This was the reason the GP was asked to review a list of their patients who had not responded and who were on the list to receive a reminder. It is notable that a reasonable number (5.11%) of subjects were deemed unsuitable to receive the letter from the GP suggesting that sending out GP-personalised letters without having a GP review in advance might be inappropriate.

As part of the ASCEND randomised controlled trial in England (a national trial designed to reduce the social gradient in uptake), investigators sought permission from general practices to allow the practice name to be added to the standard BCSP pre-invitation letter (Wardle *et al*, 2015; Raine *et al*, 2016). In all, 80% of practices agreed. An increase in uptake of 0.7 percentage points was reported (58.2% *vs* 57.5%). The authors concluded that, given the willingness shown by GPs to endorse the BCSP, the small one-off cost incurred to modify the standard invitation letter and the overall increase in uptake, the BCSP should consider adding the GP endorsement to the screening invitation letter. The London BCSP Hub have implemented the GP endorsement.

Compared with the ASCEND GP-endorsed pre-invitation intervention (Raine *et al*, 2016), the PEARL project has demonstrated a greater impact on uptake, possibly because subjects received a second reminder that appeared to have been sent by their own GP. Consideration does need to be given to the feasibility of PEARL compared with ASCEND. The ASCEND approach requires only that a GP practice agrees once a year for the endorsement to be added to the standard letters. In contrast, PEARL required continuous engagement from GP practices and created additional work for the Hub. The benefits of the PEARL approach are clear by the increase in uptake, whether this is sustainable across the country is questionable when considering the pressures that GPs face.

We have developed a robust and sustainable method to send GP-endorsed letters to non-responders. A GP-endorsed second reminder letter significantly increased uptake, by about 3 percentage points, both as a proportion of all invitees or only those who had not returned a test kit by the index date. The extra work required for the Hub and GPs to support the PEARL intervention should be evaluated and recommendations made on the feasibility of rolling out this process nationally within BCSP. The process would require refinement before being rolled out on a larger scale.

## Figures and Tables

**Table 1:** Characteristics of invitees in PEARL and comparison practices

**Factor**	**Category**	**No. (%) in PEARL practices**	**No. (%) in practices matched to PEARL practices**	**No. (%) in all non-PEARL practices**
Age	⩽65	6096 (47)	6210 (52)	576 764 (46)
	66+	6782 (53)	5648 (48)	671 925 (54)
Sex	Male	6642 (52)	5934 (50)	607 256 (49)
	Female	6236 (48)	5924 (50)	641 433 (51)
Screen type	Prevalent	6373 (49)	6295 (53)	524 744 (42)
	Incident	6505 (51)	5563 (47)	723 945 (58)
IMD quintile	1 (most deprived)	3759 (29)	2724 (23)	73 797 (6)
	2	3033 (24)	3267 (28)	182 497 (15)
	3	2836 (22)	2251 (19)	291 373 (24)
	4	2190 (17)	1218 (10)	310 743 (25)
	5 (least deprived)	996 (8)	2332 (20)	380 078 (31)
	NK	64	66	10 201
All	N/A	12 878	11 858	1 248 689

Abbreviations: IMD=index of multiple deprivation; N/A=not applicable; NK = not known; PEARL = Practice Endorsed Additional Reminder Letter.

**Table 2:** Numbers (%) adequately screened on or before the index date

**Factor**	**Category**	**PEARL practices**	**Practices matched to PEARL practices**	**All non-PEARL practices**
Age	⩽65	2741 (45)	2661 (43)	308 777 (54)
	66+	3662 (54)	2891 (51)	415 329 (62)
Sex	Male	3070 (46)	2606 (44)	332 011 (55)
	Female	3333 (53)	2946 (50)	392 095 (61)
Screen type	Prevalent	1279 (20)	1189 (19)	129 433 (25)
	Incident	5124 (79)	4363 (78)	594 673 (82)
IMD quintile	1 (most deprived)	1539 (41)	1029 (38)	32 010 (43)
	2	1391 (46)	1436 (44)	92 912 (51)
	3	1515 (53)	1035 (46)	165 219 (57)
	4	1310 (60)	662 (54)	187 682 (60)
	5 (least deprived)	610 (61)	1358 (58)	240 334 (63)
	NK	38 (59)	32 (48)	5949 (60)
All	N/A	6403 (50)	5552 (47)	724 106 (58)

Abbreviations: IMD=index of multiple deprivation; N/A=not applicable; NK = not known; PEARL = Practice Endorsed Additional Reminder Letter.

**Table 3:** Effect of intervention on participation in PEARL practices

**Population**	**Observed/expected participating subjects in PEARL practices**	**No. (%) participating**	**O/E** **(95% CI)**	**Significance**
All invitees	Observed	6914 (54)	1.06 (1.03–1.09)	*P*<0.001
	Expected	6543 (51)		
Those not returning on or before index date	Observed	362 (7)	2.17 (1.96–2.40)	*P*<0.001
	Expected	167 (3)		

Abbreviations: CI=confidence interval; O/E = Observed/Expected; PEARL = Practice Endorsed Additional Reminder Letter.

**Table 4:** Effect of intervention on participation from the direct comparison with matched practices

**Population**	**Study group**	**No. (%) participating**	**RR** **(95% CI)**	**Significance**
All invitees	Comparison practices	5861/11 858 (49)	1.00 (−)	*P*<0.001
	PEARL practices	6914/12 878 (54)	1.08 (1.05–1.11)	
Those not returning on or before index date	Comparison practices	144/5417 (3)	1.00 (−)	*P*<0.001
	PEARL practices	362/5535 (7)	2.18 (1.79–2.66)	

Abbreviations: CI=confidence interval; PEARL = Practice Endorsed Additional Reminder Letter; RR = relative risk.

**Table 5:** Effect of intervention on participation in subgroups of PEARL practices by age, sex, screen type, and IMD quintile

		**Observed and expected numbers adequately screened, and total subjects**			**Significance**	
**Population**	**Demographic subgroup**	**Observed (%)**	**Expected (%)**	**O/E (95% CI)Total**		
All subjects	Age ⩽65 years	2987 (49)	2966 (49)	6096	1.01 (0.97–1.05)	*P*=0.6
	Age >65 years	3927 (58)	3577 (53)	6782	1.10 (1.06–1.14)	*P*<0.001
	Males	3347 (50)	3184 (48)	6642	1.05 (1.01–1.09)	*P*=0.005
	Females	3567 (57)	3359 (54)	6236	1.06 (1.02–1.10)	*P*<0.001
	Prevalent screen	1465 (23)	1345 (21)	6373	1.09 (1.03–1.15)	*P*=0.001
	Incident screen	5449 (84)	5197 (80)	6505	1.05 (1.02–1.08)	*P*<0.001
	Most deprived quintile	1657 (44)	1578 (42)	3759	1.05 (1.00–1.11)	*P*=0.05
	Other four quintiles	5218 (58)	4933 (54)	9055	1.06 (1.03–1.09)	*P*<0.001
Subjects not returning an	Age ⩽65 years	181 (6)	104 (4)	2949	1.74 (1.49–2.03)	*P*<0.001
adequate kit by the index date	Age >65 years	181 (7)	63 (2)	2586	2.87 (2.42–3.41)	*P*<0.001
	Males	195 (6)	95 (3)	3058	2.05 (1.78–2.36)	*P*<0.001
	Females	167 (7)	73 (3)	2477	2.29 (1.96–2.67)	*P*<0.001
	Prevalent screen	157 (4)	70 (2)	4484	2.24 (1.90–2.63)	*P*<0.001
	Incident screen	205 (20)	97 (9)	1051	2.11 (1.83–2.43)	*P*<0.001
	Most deprived quintile	87 (5)	45 (2)	1919	1.93 (1.56–2.39)	*P*<0.001
	Other four quintiles	274 (8)	122 (3)	3593	2.25 (1.99–2.54)	*P*<0.001

Abbreviations: CI=confidence interval; IMD = index of multiple deprivation; O/E = Observed/Expected; PEARL= Practice Endorsed Additional Reminder Letter.
