# Supplementary material for: GP participation in increasing uptake in a national bowel cancer screening programme: the PEARL project
Source: Br J Cancer. 2017 May 18;116(12):1551–7. doi: 10.1038/bjc.2017.129 (PMC5518858; doi:10.1038/bjc.2017.129)
Supplement: Supplementary Appendix A [file bjc2017129x1.doc]

1. **The PEARL (Practice Endorsed Additional Reminder Letter) Project: a Bowel Cancer Screening Pilot**

**Information and Consent Form**

The PEARL project has been developed to test and establish a simple and practical means for GP practices to encourage their patient population to participate in the NHS Bowel Cancer Screening Programme (BCSP). The Pilot originated as a joint initiative of the Wessex Strategic Clinical Networks (SCN) and NHS Bowel Cancer Screening Southern Programme Hub. It is designed to improve participation in the Programme, whilst not deviating from ‘informed choice’ principles.

Non-participation in bowel cancer screening may be defined as:

1. *Non-response following a reminder letter (reminders are sent 28 days after the test kit is sent).*
2. *Commencement of screening but failure to complete a repeat test (or tests) to provide a definitive outcome of ‘normal’ or ‘abnormal’ (i.e. first test kit completed with unclear results).*

**This Pilot will be targeted at subjects who, after one month, have not responded to the usual BCSP reminder letter.**

Practical issues that currently inhibit effective primary care endorsement of the BCSP:

1. Consortia or area-wide targeted intervention, including selection according to deprivation status, age, sex and postcode.
2. Timely check of non-response status just prior to mailing an endorsement letter.
3. Mailing logistics – printing, mailing and equipment and staff resource.
4. Mailing costs – expense compared with discount mailing under a bulk mail contract.
5. The need to repeat the endorsement at regular intervals in order to reach all of the target population over a two-year BCSP cycle.
6. Ensuring that the endorsement letter is accurate and appropriate:
   - 1. It accurately describes the current BCSP process and services provided by the Hub
     2. It uses correct addresses and Hub telephone number
     3. It adopts the principles of informed choice and cannot be considered coercive.
7. Ensuring that repeat interventions are undertaken in a timely manner.
8. Cost of multiple practices undertaking similar processes and not utilising the economies of scale available through the BCSP Hub structure.
9. The time and effort needed to review and exclude individuals from the target population who should not be encouraged to participate because of their current health status.

**The BCSP is unable to take comorbidity into account when inviting subjects for screening but GP involvement will enable ‘GP-endorsed’ reminder letters to be targeted appropriately.**

**How the Pilot will work:**

**Preparation**

1. A BCSP Southern Hub contact will have been identified and their postal address, NHS email address and telephone number made available to the General Practice.
2. The General Practice and Hub will sign the Consent Form (below) that explains that data will be shared between the Hub and the General Practice, how the data will be used, how confidentiality will be maintained and the maximum period for which data will be retained.
3. The General Practice will identify two contacts for the period of the Pilot and will provide the Hub with postal addresses, NHS email addresses and telephone numbers for those contacts.
4. In discussion with the General Practice (either directly or through another NHS co-ordinating group) the criteria for a GP-endorsed reminder letter will be agreed between the General Practice and the Hub:
5. The target population will typically be subjects who have not responded to an invitation to be screened following the usual BCSP reminder letter (the cohort will be those who between one and two months after the date of the usual reminder letter, have not responded).
6. The frequency of exchange of subject lists (typically at monthly intervals)
7. The likely size of the target population for the endorsement period will be determined by the Hub and the General Practice.
8. An example list on a Microsoft Excel spread sheet will be forwarded to the specified General Practice contact.
9. The content of a GP-endorsed reminder letter will be agreed between the General Practice and the Hub. The content of the letter will be checked by the BCSP National Office to ensure that it meets informed choice requirements.
10. The General Practice will provide the Hub with an electronic copy of the practice’s headed paper plus the electronic signature of the GP working with the Pilot. The Hub will then produce a specimen mail merged letter for formal checking by the General Practice and the Hub.
11. Commencement and completion dates will be formally agreed with the General Practice.

**GP Vetting required**

1. Every month the Hub will prepare a list of subjects who have not returned their bowel cancer screening kits, using the Bowel Cancer Screening System (BCSS) and will forward the list by secure NHS email to the General Practice contact.
2. The General Practice will review the list of subjects, mark on the list any subject who must be excluded and return the list to the Hub contact.
3. The Hub will check the list against current BCSP data and exclude subjects who have responded since the list was sent to practices, have changed General Practice or who have died.
4. The Hub will revise the list, remove all excluded subjects and prepare mail merge letters using the letter template agreed by the General Practice.
5. All letters will be sent from the Hub by second class using the Royal Mail postal system.
6. The process will be repeated every month until the Pilot is complete.

**Monitoring**

1. The Hub will monitor the response to the GP-endorsed reminder letters, compare it with a control population that has similar geodemographic characteristics and will share the data with the General Practice.
2. The General Practice will provide the Hub with information and observations related to the Pilot from patients and from practice colleagues, to be collated following the Pilot period.
3. A summary document and presentation outlining the Pilot findings will be prepared by the Hub once the Pilot is complete and sufficient time has elapsed to enable reliable data to be extracted from BCSS.

**PEARL Bowel Cancer Screening Pilot**

**Confidentiality Agreement and Data Exchange Consent Form 2015/16**

**Service Provider**

NHS Bowel Cancer Screening Southern Programme Hub

20 Priestley Road

Surrey Research Park

Guildford

Surrey GU2 7YS

Free Helpline no. 0800 707 6060

**Pilot Lead**

BCSP Southern Hub Director

Sally Benton:

The Bowel Cancer Screening Southern Programme Hub is part of the Royal Surrey County Hospital NHS Foundation Trust (RSCH) and all staff employed by the Hub hold a current RSCH NHS employment contract.

The Hub conforms to the NHS Information Governance requirements. All members of staff receive annual information governance training and sign annually to the effect that they have read, understood and conform to the requirements of the NHS Information Governance.

**Hub Declaration**

The PEARL Bowel Cancer Screening Pilot will be conducted as described in this document under the leadership of the Hub Director. All documents (letterheads, stationery, scanned signatures) provided to the Hub by General Practices for the purposes of providing endorsement of the BCSP, and participation therein, will be used only for that purpose and will be destroyed on completion of the Pilot. No data produced by the Hub or provided by the General Practice will be shared in an identifiable form with individuals outside the Hub or General Practice.

**Security**

*Only anonymised data will be shared outside the Hub and participating General Practices. On completion of the Pilot only anonymised data will be retained and all names/data will be erased/destroyed. Only NHS.net email accounts will be used for data exchange.*

**PEARL Bowel Cancer Screening Pilot**

**Confidentiality Agreement and Data Exchange Consent Form 2015/16**

Name: Sally C Benton FRCPath

Role and job title: BCSP Southern Hub Director

Signature Date

**General Practice details**

Name

Address

Telephone number

Senior Partner/ GP lead

NHS.net email address

Telephone number

**Pilot contact**

Name

NHS.net email address

Telephone number

This General Practice is a participant in a Pilot to encourage participation in the Bowel Cancer Screening Programme.

**Practice declaration**

The PEARL Bowel Cancer Screening Pilot will be conducted as described in this document with support from the General Practice Senior Partner/Lead. The General Practice will provide materials (letterheads, stationery, scanned signatures) to the Hub and will undertake, if required, in the agreed Pilot to vet lists of subjects eligible for screening for the purposes of providing endorsement of the Bowel Cancer Screening Programme. No Data produced by the Hub or provided by the General Practice will be shared in an identifiable form with individuals outside the Hub or General Practice.

Signature

General Practice Senior Partner/Lead

Role and Title

Date
