# Supplementary material for: GP participation in increasing uptake in a national bowel cancer screening programme: the PEARL project
Source: Br J Cancer. 2017 May 18;116(12):1551–7. doi: 10.1038/bjc.2017.129 (PMC5518858; doi:10.1038/bjc.2017.129)

1. **PEARL letter template**

Dear

The NHS Bowel Cancer Screening Programme sent you a screening test kit a few weeks ago that has not yet been received for testing.

Some of our patients make a choice not to do the test but others are undecided or worry about doing it. The practice decided that we should write to you and let you know that we believe it is a very valuable test that has saved many lives.

Bowel cancer is very common and, if it is found early by using this test, it can usually be completely removed. **Our practice recommends that you consider doing this simple test because it could save your life.**

If you no longer have your test kit then phone the Screening Hub **0800 707 6060** (free for landlines) and ask for a replacement kit.

If you already have symptoms that might be caused by bowel cancer then please make an appointment to see one of us. The common symptoms of bowel cancer are listed below – we all need to be aware of them:

- Repeated and unexplained bleeding from your bottom or blood in your stool (poo).
- A persistent change in ‘bowel habit’ (going more often or frequent diarrhoea).
- Severe and continuing pain in your tummy.
- A lump in your tummy.
- Recent and unexplained loss of weight.
- Unexplained tiredness.

These symptoms are not usually due to cancer, but if you have had any of them for more than 4 weeks then please make an appointment with us.

Yours sincerely,


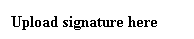

Supplement: Supplementary Appendix B [file bjc2017129x2.doc]
