# Supplementary material for: GP participation in increasing uptake in a national bowel cancer screening programme: the PEARL project
Source: Br J Cancer. 2017 May 18;116(12):1551–7. doi: 10.1038/bjc.2017.129 (PMC5518858; doi:10.1038/bjc.2017.129)
Supplement: Supplementary Appendix C [file bjc2017129x3.doc]

1. **Calculation of expected numbers adequately screened**

As noted above, we first estimated the associations of age, sex, episode type, IMD quintile and prior prevalent participation with the probability of being adequately screened, using logistic regression, within the non-PEARL registered practices. The logistic regression estimates and their standard errors are shown in Table A1. This gives a logistic regression equation


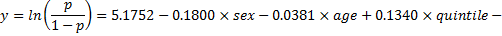


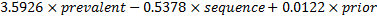


Where p is the probability of being adequately screened, sex is coded as 0=female, 1=male, prevalent is coded 0=incident screen, 1=prevalent screen, episode sequence runs from 1 (prevalent) to 8, and prior refers to the percent prevalent episode participation prior to the study. After a little algebra, this implies that the estimated probability of being adequately screened is


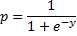


The variance of y is calculated using the covariance matrix of the estimated logistic regression coefficients and the variance of p using the approximation for transformed data:


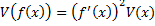


where f’ denotes the derivative of f. Thus


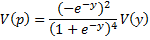


Then, for every subject in the PEARL-registered practices, we calculate p, the expected probability of being adequately screened, based on the five covariates. The average p multiplied by 12,878 gives the expected number of subjects being adequately screened in the PEARL-registered practices, standardised for age, sex, episode sequence, IMD quintile and prior prevalent participation rates. This is then compared with the observed participation in the PEARL-registered practices, with significance testing taking account of variability in the expected number (V(p)) as well as the observed.

For the estimation of standardised expected numbers adequately screened among those who had not returned a kit by the index date, we used the same procedure, but first re-estimated the logistic regression coefficients in the non-PEARL registered practices for this subgroup only. The logistic regression coefficients and their standard errors are shown in Table A2.

| Table A1. Logistic regression coefficients for being adequately screened, calculated from the non-PEARL registered practice data | | |
| --- | --- | --- |
| Factor | Logistic regression coefficient | Standard Error |
| Constant | 5.1752 | 0.0528 |
| Sex (male) | -0.1800 | 0.0048 |
| Age | -0.0381 | 0.0008 |
| IMD quintile | 0.1340 | 0.0020 |
| Prevalent screen | -3.5926 | 0.0064 |
| Episode sequence | -0.5378 | 0.0036 |
| Prior prevalent participation | 0.0122 | 0.0005 |

| Table A2. Logistic regression coefficients for being adequately screened, calculated from the non-PEARL registered practice data, in those not adequately screened by the time of the index date | | |
| --- | --- | --- |
| Factor | Logistic regression coefficient | Standard Error |
| Constant | 0.5945 | 0.1679 |
| Sex (male) | 0.0607 | 0.0159 |
| Age | -0.0326 | 0.0026 |
| IMD quintile | 0.1382 | 0.0067 |
| Prevalent screen | -2.3266 | 0.0187 |
| Episode sequence | -0.3940 | 0.0119 |
| Prior prevalent participation | 0.0058 | 0.0015 |
